# Supplementary figures and images for: Cryo-EM reveals an unprecedented binding site for NaV1.7 inhibitors enabling rational design of potent hybrid inhibitors
Source: eLife. 2023 Mar 28;12:e84151. doi: 10.7554/eLife.84151 (PMC10112885; doi:10.7554/eLife.84151)

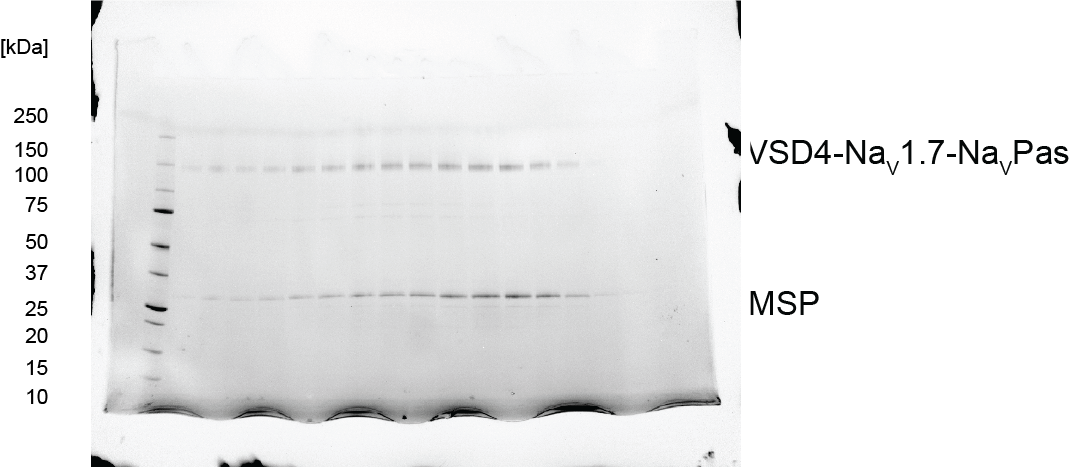

Supplement: Figure 1—figure supplement 1—source data 1. [file elife-84151-fig1-figsupp1-data1.zip › Supplementary Figure1-Source Data/Untitled-1.png]

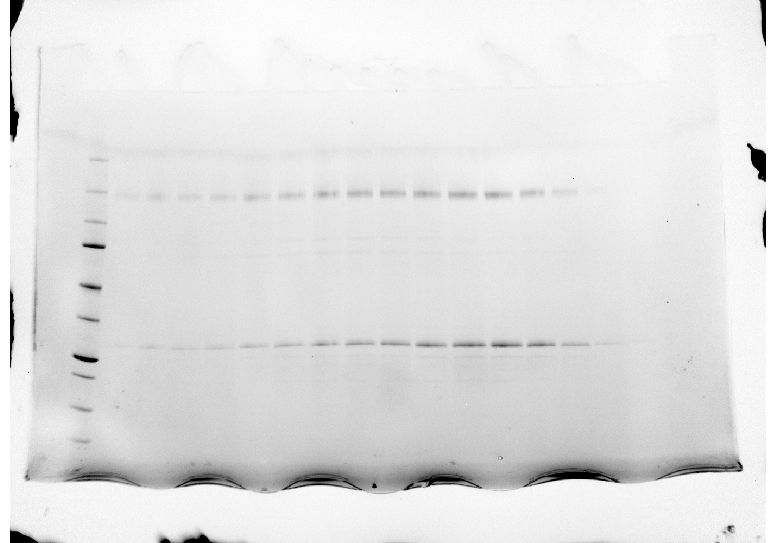

Supplement: Figure 1—figure supplement 1—source data 1. [file elife-84151-fig1-figsupp1-data1.zip › Supplementary Figure1-Source Data/20201022-gel02-NavPas-nanodisc-reconst-2nd-Strep-uSup6.jpeg]
