# Supplementary material for: Cryo-EM reveals an unprecedented binding site for NaV1.7 inhibitors enabling rational design of potent hybrid inhibitors
Source: eLife. 2023 Mar 28;12:e84151. doi: 10.7554/eLife.84151 (PMC10112885; doi:10.7554/eLife.84151)

**Supporting File**

**CryoEM reveals unprecedented binding site for Na_V_1.7 inhibitors enabling rational design of potent hybrid inhibitors**

Marc Kschonsak^1*^, Christine C. Jao^1*^, Christopher P. Arthur^1^, Alexis L. Rohou^1^, Philippe Bergeron^2^, Daniel Ortwine^2^, Steven J. McKerrall^2^, David H. Hackos^3^, Lunbin Deng^3^, Jun Chen^4^, Tianbo Li^4^, Peter S. Dragovich^2^, Matthew Volgraf^2^, Matthew R. Wright^5^, Jian Payandeh^1,#^, Claudio Ciferri^1,#^, John C. Tellis^2,#^

^1^Genentech Inc., Structural Biology; South San Francisco, CA, United States.

^2^Genentech Inc., Discovery Chemistry; South San Francisco, CA, United States.

^3^Genentech Inc., Neuroscience; South San Francisco, CA, United States.

^4^Genentech Inc., Biochemical and Cellular Pharmacology; South San Francisco, CA, United States.

^5^Genentech Inc., Drug Metabolism and Pharmacokinetics; South San Francisco, CA, United States.

^#^Corresponding authors. Email: jpayandeh@exelixis.com (J.P); ciferri.claudio@gene.com (C.C.); tellis.john@gene.com (J.C.T.).

**Supplementary File 1A. Cryo-EM data collection, refinement and validation statistics.**

|  | **GNE-3565** VSD4-Na_V_1.7-Na_V_Pas complex | **GDC-0310** VSD4-Na_V_1.7-Na_V_Pas complex | **GNE-1305** VSD4-Na_V_1.7-Na_V_Pas complex | **GNE-9296** VSD4-Na_V_1.7-Na_V_Pas-DC1a complex |
| --- | --- | --- | --- | --- |
| **Data Collection** |  |  |  |  |
| Magnification | 105,000x | 105,000x | 165,000x | 165,000x |
| Voltage (kV) | 300 | 300 | 300 | 300 |
| Electron exposure (e/Å^2^) | 64 | 64 | 64 | 48 |
| Defocus range (µm) | 0.5-1.5 | 0.5-1.5 | 0.5-1.5 | 0.5-1.5 |
| Pixel size (Å) | 0.838 | 0.838 | 0.838 | 0.824 |
| Symmetry imposed | C1 | C1 | C1 | C1 |
| Initial particle images | 3,686,837 | 2,253.983 | 2,741,402 | 1,774,062 |
| Final particle images | 964,572 | 795,792 | 1,201,168 | 826,525 |
| Map resolution (Å) overall | 2.9 | 2.5 | 2.2 | 3.1 |
| FSC threshold | 0.143 | 0.143 | 0.143 | 0.143 |
| Map resolution range (Å) | 2.7-33.5 | 2.3-33.5 | 2.1-33.5 | 3.3-33.0 |
| **Refinement** |  |  |  |  |
| Initial models used (PDB code) | 6NT3 | 6NT3 | 6NT3 | 6NT3 |
| Model resolution (Å) | 2.9 | 1.9 | 1.7 | 2.5 |
| FSC threshold | 0.5 | 0.5 | 0.5 | 0.5 |
| Model composition |  |  |  |  |
| Non-hydrogen atoms | 9396 | 9557 | 9616 | 9734 |
| Protein residues | 1115 | 1115 | 1117 | 1159 |
| Waters | 0 | 78 | 111 | 0 |
| Ligands | 13 | 13 | 14 | 13 |
| B factors (Å^2^) |  |  |  |  |
| Protein | 25.62 | 12.42 | 6.27 | 24.05 |
| Ligand | 35.81 | 15.79 | 10.33 | 40.62 |
| Water | N/A | 8.11 | 6.54 | N/A |
| R.m.s. deviations |  |  |  |  |
| Bond lengths (Å) | 0.004 | 0.005 | 0.005 | 0.005 |
| Bond angles (°) | 0.931 | 1.018 | 0.994 | 0.941 |
| Validation |  |  |  |  |
| MolProbity score | 1.30 | 1.19 | 1.14 | 1.38 |
| Clashscore | 5.47 | 4.09 | 3.51 | 6.42 |
| Poor rotamers (%) | 0.10 | 0.20 | 0.61 | 0.00 |
| Ramachandran Plot |  |  |  |  |
| Favored (%) | 98.45 | 98.27 | 98.19 | 97.90 |
| Allowed (%) | 1.55 | 1.73 | 1.81 | 2.10 |
| Disallowed (%) | 0.00 | 0.00 | 0.00 | 0.00 |
|  |  |  |  |  |

**Chemical compound synthesis and characterization**

**General Considerations**

All commercial solvents and reagents were used without additional purification unless indicated otherwise. ^1^H NMR spectra were measured on Bruker Avance III 300, 400, or 500 MHz spectrometers. Chemical shifts (in ppm) were referenced to tetramethylsilane as an internal standard (δ = 0 ppm). Reaction progress was monitored by either a Shimadzu LCMS/UV system with an LC-30 AD solvent pump, Sil-30 AC autosampler, 2020 MS, SPDM30A UV detector, and CTO-20A column oven, using 2−98% acetonitrile/0.1% formic acid (or 0.01% ammonia) over 2.5 min or a Waters Acquity LCMS system using 2−98% acetonitrile/0.1% formic acid (or 0.1% ammonia) over 2 min. Flash column chromatography purifications were performed using a Teledyne Isco Combiflash Rf and Silicycle HP columns. Reverse-phase purification was done on a Phenomenex Gemini-NX C18 (30 × 100 mm, 5 μm) with a gradient of 5−95% acetonitrile/water (with 0.1% NH4OH or 0.1% formic acid) at 60 mL/min over 10 min. Preparative SFC separations were carried out on a PIC Solutions instrument, with conditions specified in the Experimental Section. High-resolution mass spectrometry (HRMS) of final compounds was obtained on a Thermo UHPLC/ QE with a Thermo-Q Exactive mass spectrometry detector using ESI ionization, following elution on an Acquity BEH C18 stationary phase (2.1 mm × 50 mm; 1.7 μm particle size) using a gradient of water/ acetonitrile (3−97% over 7 min with 0.1% formic acid in both phases). Unless stated otherwise, analytical purity was >95% as determined by LCMS using UV 254 nm detection

**Compound 2 / GNE-9296**

**5-chloro-4-(cyclopentylmethoxy)-*N*-((4-(((1*S*,2*S*)-2-(dimethylamino)cyclohexyl)amino)-2-fluorophenyl)sulfonyl)-2-fluorobenzamide**

**Step** **1:**

**4-(((1*S*,2*S*)-2-(dimethylamino)cyclohexyl)amino)-2-fluorobenzenesulfonamide**

A solution of (1*S*,2*S*)-*N*^1^,*N*^1^-dimethylcyclohexane-1,2-diamine (162 mg, 1.1 mmol), 2,4-difluorobenzenesulfonamide (200 mg, 1.0 mmol) and DIPEA (0.3 mL, 1.7 mmol) in DMSO (3 mL) was stirred at 80 ^o^C for 16 h. After cooling to room temperature, the reaction was diluted with EtOAc (100 mL), and washed with brine (50 mL x 5). The organic lawyer was dried over anhydrous Na_2_SO_4_, filtered and concentrated in vacuo. The crude residue was purified by silica gel chromatography (solvent gradient: 0 - 10% EtOAc in petroleum ether) to afford the title compound (60 mg, 0.19 mmol) as yellow oil.

LCMS (ESI) m/z: 316.1 [M+H]^+^.

**Step** **2:**

**tert-butyl 5-chloro-4-(cyclopentylmethoxy)-2-fluorobenzoate**

A mixture of tert-butyl 5-chloro-2,4-difluorobenzoate (548 g, 2.20 mol), cyclopentylmethanol (200 g, 2.00 mol) and Cs_2_CO_3_ (1.31 kg, 4.00 mol) in DMSO (3.28 L) was stirred at 80 °C for 5 hrs. The mixture was filtered and filtrate was diluted with EtOAc (3.00 L), washed with water (3.00 L x 3) and brine (1000 mL). The resultant organic layer was dried over Na_2_SO_4_, filtered, concentrated in vacuo. The residue was purified by column chromatography (SiO_2_, Petroleum ether/Ethyl acetate = 100/1 to 50/1). The title compound (0.50 kg, crude) was obtained as light yellow oil.

**Step 3:**

**5-chloro-4-(cyclopentylmethoxy)-2-fluorobenzoic acid**

To a solution of *tert*-butyl 5-chloro-4-(cyclopentylmethoxy)-2-fluorobenzoate (327 g, 994 mmol) in DCM (654 mL) was added TFA (654 mL) at 25 °C. The mixture was stirred at 25 °C for 24 hrs. The reaction mixture was filtered and concentrated under reduced pressure to give a residue that was triturated with 1/1 MTBE/Petroleum ether at 25 °C for 30 mins. The title compound was obtained by filtration as a white solid (83.7 g, 30.5% yield).

^1^H NMR (400 MHz, CDCl_3_) *δ* 8.04 (d, *J* = 7.6 Hz, 1H), 6.70 (d, *J* = 12.4 Hz, 1H), 3.96 (d, *J* = 6.8 Hz, 2H), 2.42-2.50 (m 1H), 1.86-1.93 (m, 2H), 1.75-1.58 (m, 4H), 1.48-1.35 (m, 2H).

**Step 4:**

**5-chloro-4-(cyclopentylmethoxy)-*N*-((4-(((1*S*,2*S*)-2-(dimethylamino)cyclohexyl)amino)-2-fluorophenyl)sulfonyl)-2-fluorobenzamide**

To a solution of 4-(((1*S*,2*S*)-2-(dimethylamino)cyclohexyl)amino)-2-fluorobenzenesulfonamide (80 mg, 0.24 mmol) and DMAP (59 mg, 0.48 mmol) in DCM (2 mL) was added EDCI (51 mg, 0.26 mmol) and 5-chloro-4-(cyclopentylmethoxy)-2-fluoro-benzoic acid (72 mg, 0.26 mmol). The reaction was stirred at room temperature for 2 h. The reaction was quenched with 10% aqueous citric acid (5 mL). The reaction was diluted with water (10 mL) and extracted with DCM (10 mL x 3). The combined organic layers were dried over anhydrous Na_2_SO_4_, filtered and concentrated in vacuo. The crude residue was purified by silica gel chromatography (solvent gradient: 0 - 5% MeOH in DCM) to afford the title compound (40 mg, 28%) as a white solid.

^1^H NMR (400 MHz, DMSO-*d*_6_) δ 7.75 (d, *J*= 7.6 Hz, 1H), 7.55 - 7.48 (m, 1H), 6.94 (d, *J*= 12.4 Hz, 1H), 6.50 (s, 1H), 6.47 (s, 1H), 6.07 (d, *J*= 10.0 Hz, 1H), 3.94 (d, *J*= 6.8 Hz, 2H), 3.79 - 3.64 (m, 1H), 3.15 - 3.05 (m, 1H), 2.66 (s, 6H), 2.35 - 2.25 (m, 1H), 2.09 - 1.94 (m, 2H), 1.84 - 1.69 (m, 3H), 1.63 - 1.14 (m, 11H).

HRMS *m/z* calcd for C_27_H_34_ClF_2_N_3_O_4_S [M+H]^+^: 570.1999. Found 570.2003.

**Compound 3**

**4-(cyclopentylmethoxy)-5-cyclopropyl-N-((4-(((1S,2S)-2-(dimethylamino)cyclohexyl)amino)-2-fluorophenyl)sulfonyl)-2-fluorobenzamide**

Following the procedure described in compound **3** and replacing 5-chloro-4-(cyclopentylmethoxy)-2-fluorobenzoic acid with 4-(cyclopentylmethoxy)-5-cyclopropyl-2-fluorobenzoic acid, the title compound was obtained as a white solid.

^1^H NMR (400 MHz, DMSO-*d*_6_) δ 7.50 (t, *J* = 8.8 Hz, 1H), 7.20 (d, *J* = 8.8 Hz, 1H), 6.65 (d, *J* = 12.0 Hz, 1H), 6.52 - 6.34 (m, 2H), 5.99 (s, 1H), 3.87 (d, *J* = 6.8 Hz, 2H), 3.72 - 3.52 (m, 1H), 3.05 - 2.81 (m, 1H), 2.61 - 2.52 (m, 6H), 2.37 - 2.26 (m, 1H), 2.07 - 1.90 (m, 3H), 1.87 - 1.71 (m, 3H), 1.66 - 1.52 (m, 5H), 1.45 - 1.30 (m, 4H), 1.29 - 1.20 (m, 1H), 1.15 - 1.05 (m, 1H), 0.91 - 0.80 (m, 2H), 0.60 - 0.45 (m, 2H).

HRMS *m/z* calcd for C_30_H_39_F_2_N_3_O_4_S [M+H]^+^: 576.2702. Found 576.2713.

**Compound 4 / GNE-1305**

**N-(6-(cyclopentylmethoxy)benzo[d]thiazol-2-yl)-4-(((1S,2S)-2-(dimethylamino)cyclohexyl)amino)-2-fluorobenzenesulfonamide**

**Step 1:**

**4-(cyclopentylmethoxy)aniline**

To a stirred solution of NaH (1.32 g, 54.98 mmol, 60% in mineral oil) in DMF (20 mL) was added 4-aminophenol (2 g, 18.33 mmol) at 0 ^o^C under nitrogen atmosphere. After stirring at 0 ^o^C for 10 min, (bromomethyl)cyclopentane (4.48 g, 27.49 mmol) was added at 0 ^o^C. The reaction was stirred at room temperature for 16 h. The reaction was quenched with water (100 mL) and extracted with EtOAc (100 mL x 3). The combined organic lawyers were washed with brine (100 mL), dried over anhydrous Na_2_SO_4_, filtered and concentrated in vacuo. The crude residue was purified by silica gel chromatography (solvent gradient: 10 - 20% EtOAc in petroleum ether) to afford the title compound (1.35 g, 39%) as black oil.

^1^H NMR (400 MHz, CDCl_3_) δ 6.77 - 6.74 (m, 2H), 6.66 - 6.63 (m, 2H), 3.76 (d, *J* = 7.2 Hz, 2H), 3.42 (s, 2H), 2.38 - 2.27 (m, 1H), 1.86 - 1.78 (m, 2H), 1.64 - 1.58 (m, 4H), 1.39 - 1.30 (m, 2H).

LCMS (ESI) m/z: 192.2 [M+H]^+^.

**Step 2:**

**6-(cyclopentylmethoxy)benzo[*d*]thiazol-2-amine**

The solution of 4-(cyclopentylmethoxy)aniline (1.30 g, 7.06 mmol) and potassium thiocyanate (685 mg, 7.06 mmol) in acetic acid (7.5 mL) was stirred at 0 ^o^C for 20 min. Bromine (0.36 mL, 7.06 mmol) in acetic acid (3.5 mL) was added slowly and keep the temperature below 10 °C. Then, the mixture was stirred at room temperature for 18 h. The reaction was filtered and the filter cake was washed with acetic acid (5 mL). The filtrate was concentrated in vacuo and the crude reside was diluted with hot water (5 mL) and basified to pH>11 with NH_3_•H_2_O. The resulting precipitate was filtered and the filter cake was washed with water (5 mL). The filter cake was diluted with DCM (20 mL), dried over anhydrous Na_2_SO_4_, filtered and concentrated in vacuo. The crude residue was purified by silica gel chromatography (solvent gradient: 0 - 14% EtOAc in petroleum ether) to afford the title compound (800 mg, 46%) as a gray solid.

^1^H NMR (400 MHz, CDCl_3_) δ 7.43 (d, *J* = 8.8 Hz, 1H), 7.13 (d, *J* = 6.0 Hz, 1H), 6.93 - 6.90 (m, 1H), 5.22 (s, 2H), 3.84 (d, *J* = 7.2 Hz, 2H), 2.41 - 2.33 (m, 1H), 1.89 - 1.81 (m, 2H), 1.68 - 1.58 (m, 4H), 1.41 - 1.33 (m, 2H).

LCMS (ESI) m/z: 249.0 [M+H]^+^.

**Step 3:**

**6-(cyclopentylmethoxy)-N-(2,4-dimethoxybenzyl)benzo[d]thiazol-2-amine**

To a stirred solution of 6-(cyclopentylmethoxy)benzo[d]thiazol-2-amine (0.71 g, 2.65 mmol) and 2,4-dimethoxybenzaldehyde (0.4 g, 2.41 mmol) in DCM (12 mL) was added TiCl(O*i*-Pr)_3_ (1.86 mL, 5.54 mmol) in one portion under nitrogen atmosphere. The solution was stirred for 10 min before the portion wise addition of NaBH(OAc)_3_ (1.53 g, 7.22 mmol) at 0 ^o^C. The reaction was stirred at room temperature for 16 h. The reaction was quenched with saturated aqueous NaHCO_3_ solution (50 mL), extracted with DCM (50 mL x 3). The combined organic lawyers were washed with brine (50 mL), dried over anhydrous Na_2_SO_4_, filtered and concentrated in vacuo. The crude residue was purified by silica gel chromatography (solvent gradient: 0 - 25% EtOAc in petroleum ether) to afford the title compound (0.53 g, 53%) as a white solid.

**Step 4:**

**N-(6-(cyclopentylmethoxy)benzo[d]thiazol-2-yl)-N-(2,4-dimethoxybenzyl)-2,4-difluorobenzenesulfonamide**

To a solution of 6-(cyclopentylmethoxy)-N-(2,4-dimethoxybenzyl)benzo[d]thiazol-2-amine (170 mg, 0.52 mmol) in THF (2 mL) was added LiHMDS (0.62 mL, 0.62 mmol, 1 M) at -78 ^o^C. The reaction was stirred for 30 min at 0 °C and a solution of 2,4-difluorobenzenesulfonylchloride (0.22 g, 1.03 mmol) in THF (2 mL) was added dropwise at -78 °C. After the addition was complete, the cooling bath was removed. The reaction mixture was stirred at room temperature for 3 h. The reaction was diluted with water (30 mL) and extracted with EtOAc (50 mL x 3). The combined organic lawyers were washed with brine (50 mL), dried over anhydrous Na_2_SO_4_, filtered and concentrated in vacuo. The crude residue was purified by silica gel chromatography (solvent gradient: 0 - 30% EtOAc in petroleum ether) to afford the title compound (160 mg, 52%) as a white solid.

**Step 5:**

**N-(6-(cyclopentylmethoxy)benzo[d]thiazol-2-yl)-N-(2,4-dimethoxybenzyl)-4-(((1S,2S)-2-(dimethylamino)cyclohexyl)amino)-2-fluorobenzenesulfonamide**

To a solution of *N*-(6-bromothiazolo[4,5-*b*]pyridin-2-yl)-4-(((1*S*,2*S*)-2-(dimethylamino)cyclohexyl)amino)-2-fluorobenzenesulfonamide (40 mg, 0.07 mmol) in DMSO (1 mL) was added DIPEA (14 mg, 0.11 mmol) and (1*S*,2*S*)-*N*_1_,*N*_1_-dimethylcyclohexane-1,2-diamine (15 mg, 0.11 mmol) at room temperature. The reaction mixture was stirred at room temperature for 20 h. The reaction was quenched with saturated aqueous NH_4_Cl (20 mL), extracted with EtOAc (30 mL x 3). The combined organic layers were washed with brine (30 mL), dried over anhydrous Na_2_SO_4_, filtered and concentrated in vacuo to afford the title compound (48 mg, crude) as yellow oil that required no further purification.

**Step 6:**

**N-(6-(cyclopentylmethoxy)benzo[d]thiazol-2-yl)-4-(((1S,2S)-2-(dimethylamino)cyclohexyl)amino)-2-fluorobenzenesulfonamide**

A solution of *N*-(6-(cyclopentylmethoxy)benzo[d]thiazol-2-yl)-*N*-(2,4-dimethoxybenzyl)-4-(((1*S*,2*S*)-2-(dimethylamino)cyclohexyl)amino)-2-fluorobenzenesulfonamide (60 mg, 0.08 mmol) in HCOOH (3 mL) was stirred at room temperature for 16 h. The mixture was concentrated in vacuo and the crude residue was purified by reverse phase chromatography (acetonitrile 35 - 65% / 0.2% HCOOH in water) to afford the title compound (2 mg, 4%) as a white solid

^1^H NMR (400 MHz, DMSO-*d_6_*) δ 7.49 (t, *J* = 8.8 Hz, 1H), 7.26 (d, *J* = 2.4 Hz, 1H), 7.16 (d, *J* = 8.8 Hz, 1H), 6.84 - 6.81 (m, 1H), 6.42 - 6.36 (m, 3H), 3.79 (d, *J* = 6.8 Hz, 2H), 3.47 - 3.40 (m, 1H), 2.82 - 2.75 (m, 1H), 2.42 (s, 6H), 2.33 - 2.24 (m, 1H), 2.02 - 1.92 (m, 2H), 1.79 - 1.71 (m, 3H), 1.61 - 1.48 (m, 5H), 1.34 - 1.20 (m, 6H).

HRMS (ESI) m/z: 547.2196 [M+H]^+^

**Compound 5**

**4-(((1S,2S)-2-(dimethylamino)cyclohexyl)amino)-2-fluoro-N-(6-(trifluoromethoxy)benzo[d]thiazol-2-yl)benzenesulfonamide**

Following the procedure described in compound **6** and replacing 4-(cyclopentylmethoxy)aniline with 4-(trifluoromethoxy)aniline, the title compound was obtained as a white solid.

^1^H NMR (400 MHz, DMSO-*d_6_*) δ 7.64 (m, 1H), 7.52 (m, 1H), 7.30 (d, *J* = 8.8 Hz, 1H), 7.11 (m, 1H), 6.44-6.37 (m, 3 H), 3.68 (m, 1H), 3.06 (m, 1H), 2.60 (s, 6 H), 2.00 (m, 2H), 1.79 (m, 1H), 1.61 (m, 1H), 1.40- 1.14 (m, 4 H).

HRMS (ESI) m/z: 533.1286 [M+H]^+^.

| Compound | Na_V_1.7 | Na_V_1.5 | Na_V_1.5/  Na_V_1.7 | Na_V_1.6 | Na_V_1.6/  Na_V_1.7 |
| --- | --- | --- | --- | --- | --- |
| 1 | 24 nM | >10 uM | **>420x** | 7.4 uM | **308x** |
| 2 (GNE-9296) | 47 nM | >10 uM | **>210x** | 55 nM | **1.2x** |
| 3 | 16 nM | 6.2 uM | **390x** | 22 nM | **1.4x** |
| 4 (GNE-1305) | 9 nM | 69 nM | **78x** | 11 nM | **1.3x** |
| 5 | 74 nM | >10 uM | **>130x** | 73 nM | **1x** |
| S1 | 110 nM | 3.2 uM | **32x** | >10 uM | **>91x** |
| S2 | 17 nM | 5.1 uM | **300x** | 82 nM | **4.8x** |
| S3 | 6 nM | 3.7 nM | **0.6x** | 40 nM | **6.6x** |
| GNE-3565 | 0.7 nM | 7.2 uM | **10,000x** | 3 nM | **4.2x** |

**Supplementary File 1B:** Na_V_ subtype selectivity for selected hybrid compounds

Generating subtype selective Na_V_1.7 inhibitors has been a longstanding challenge. Our characterization of Na_V_1.7 inhibitors focused on selectivity over Na_V_1.5 and Na_V_1.6 as representative anti-targets, where arylsulfonamides and acylsulfonamides have typically displayed differentiated selectivity patterns. More specifically, arylsulfonamides will typically show high levels of selectivity over Na_V_1.5 and low levels of selectivity over Na_V_1.6. In contrast, acylsulfonamides typically have comparably higher selectivity over Na_V_1.6, but show lower selectivity against Na_V_1.5. The studies described here primarily focused on demonstrating proof-of-concept in generating potent hybrid molecules, and did not focus on selectivity as a key endpoint. However, various molecules offer potential insights about selectivity patterns for hybrid inhibitors.

Acysulfonamide **1** demonstrates unusually high selectivity against Na_V_1.5 (>420x) and Na_V_1.6 (308x). Critically, efforts to improve potency in the acylsulfonamide series result in reduction of selectivity against these off-targets, as demonstrated by representative molecule **S3**. Selectivities in this range are more common for acylsulfonamides of high potency. Note that data for GDC-0310 is not shown because the slow binding kinetics of this molecule do not allow accurate quantitation using the Sycropatch assay method.

Hybrid molecules **1-5** show selectivity patterns most similar to arylsulfonamides, with high selectivity (typically >100x) over Na_V_1.5, but low selectivity over Na_V_1.6 (Typically 1-2x). However, structural modification of these molecules can result in significant changes in selectivity patterns, with **S1** showing high selectivity over Na_V_1.6 (>91x) but reduced selectivity over Na_V_1.5 (32x). **S2** demonstrates an ability to improve Na_V_1.6 selectivity (4.8x) while maintaining Na_V_1.5 selectivity (300x) in the hybrid scaffold. Overall, this data demonstrates that hybrid series inhibitors can achieve selectivity over both Na_V_1.5 and Na_V_1.6. Moreover, we speculate that hybrid inhibitors may offer more opportunities for selectivity than previous generation chemical matter, because molecules from this series have access to selectivity determinants in *both* the aryl- and acylsulfonamide binding pockets *simultaneously*.

HPLC chromatogram of compound **2**


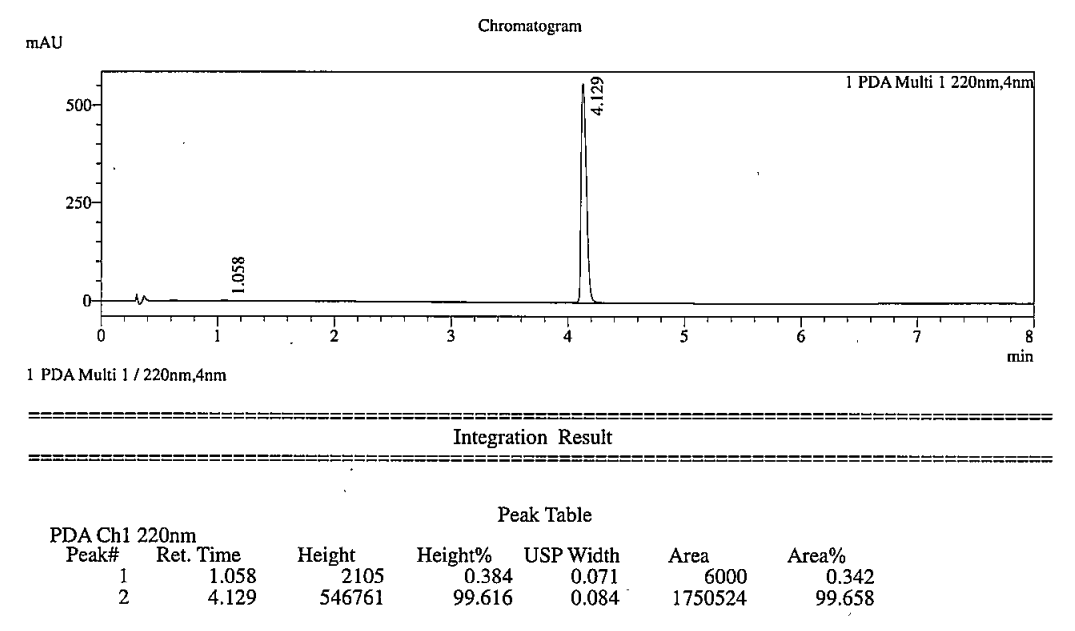


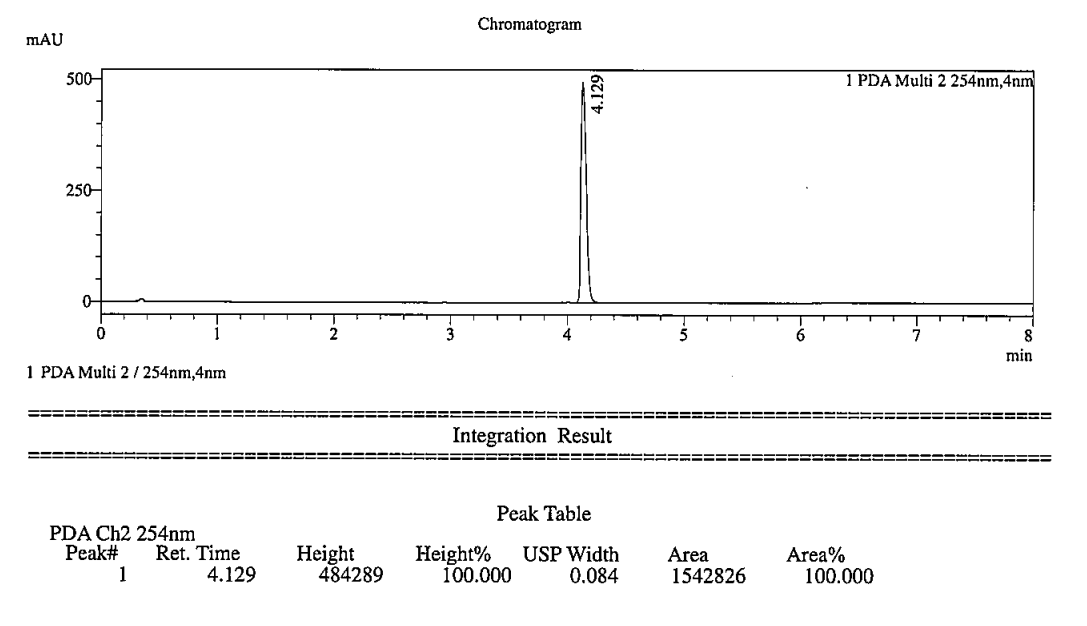


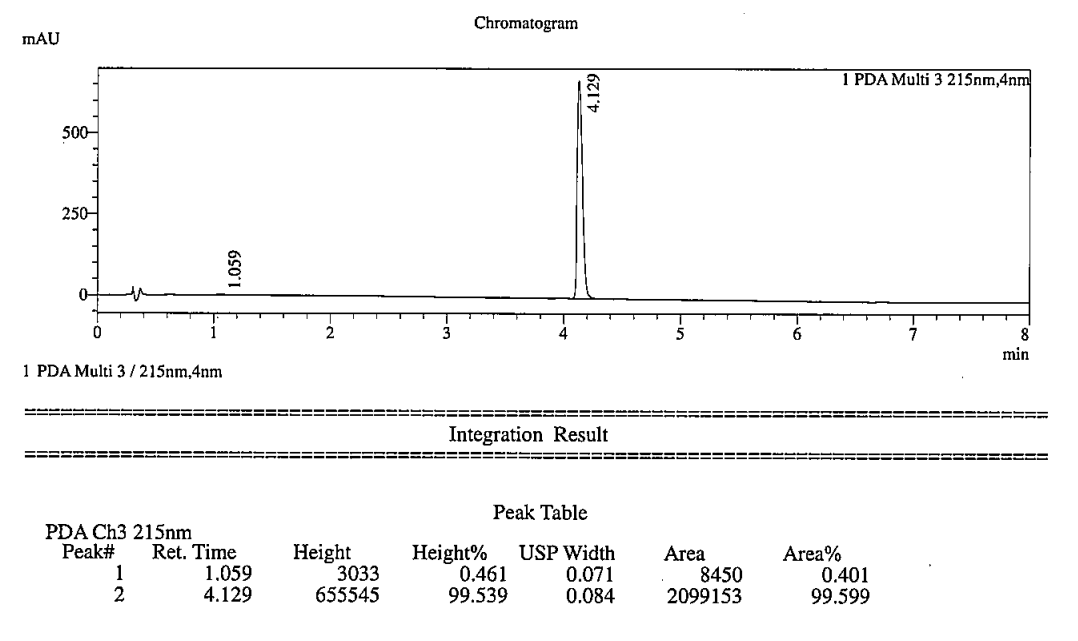


HPLC chromatogram of compound **3**


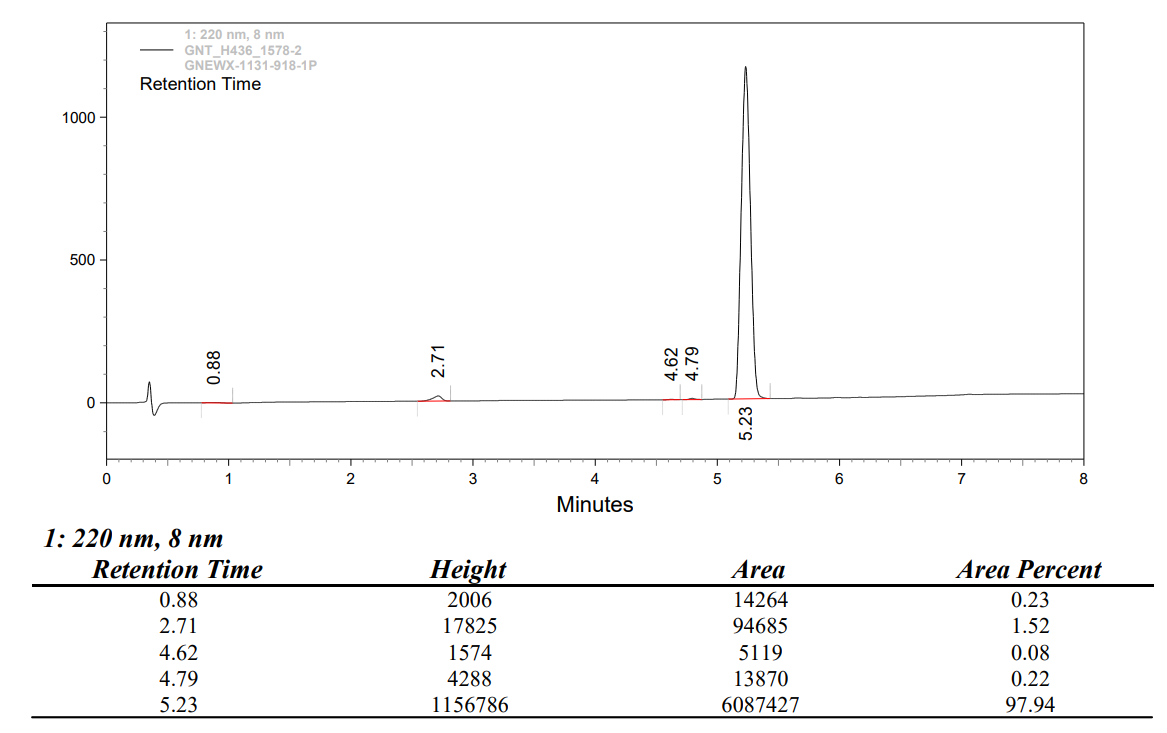


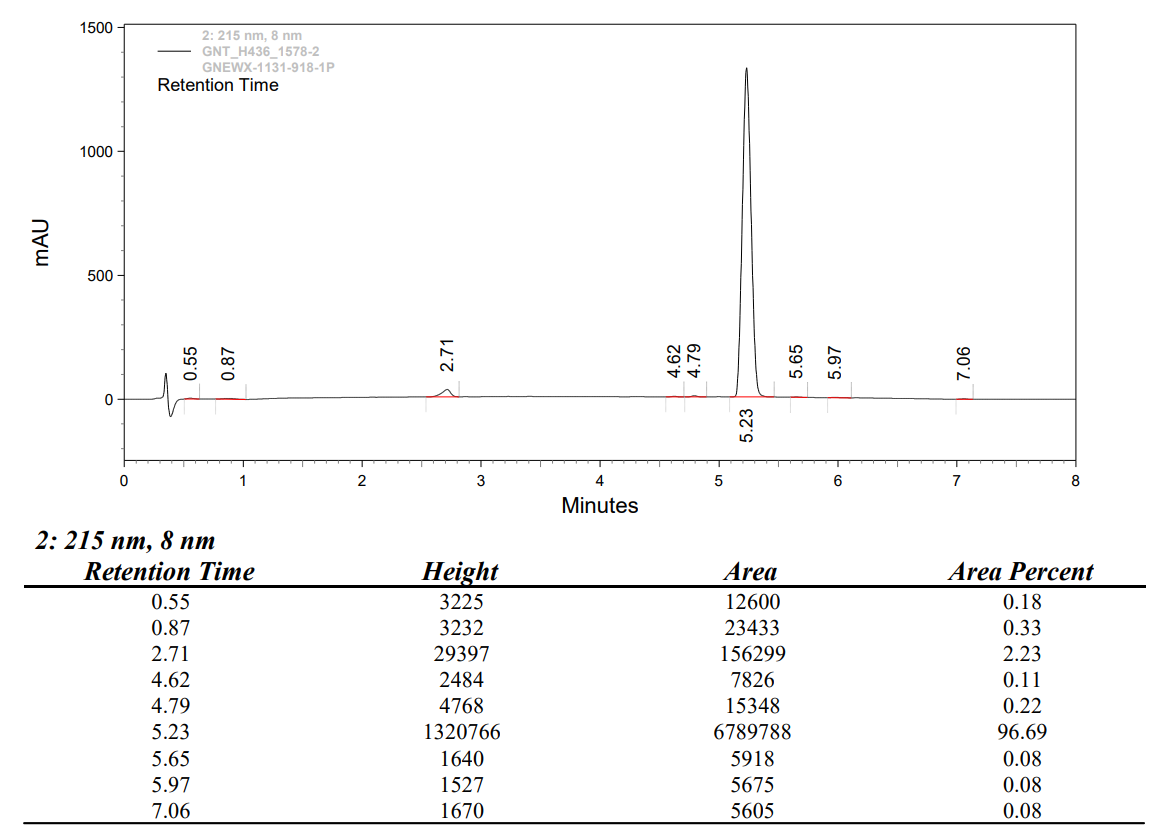


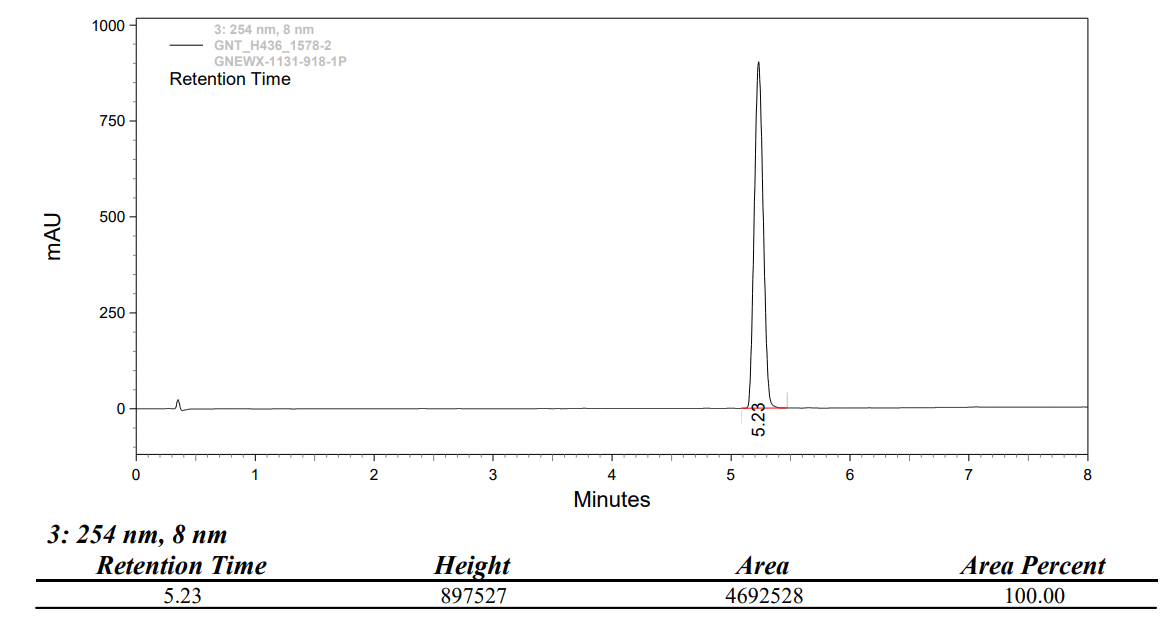


HPLC chromatogram of compound **4**


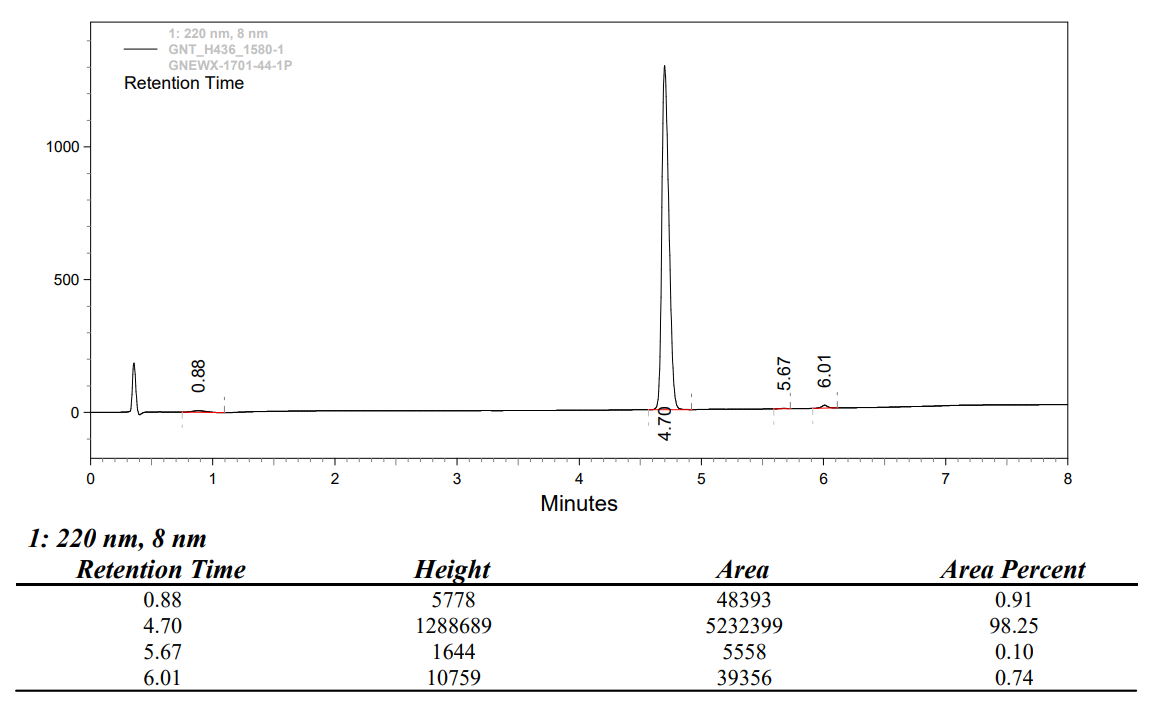


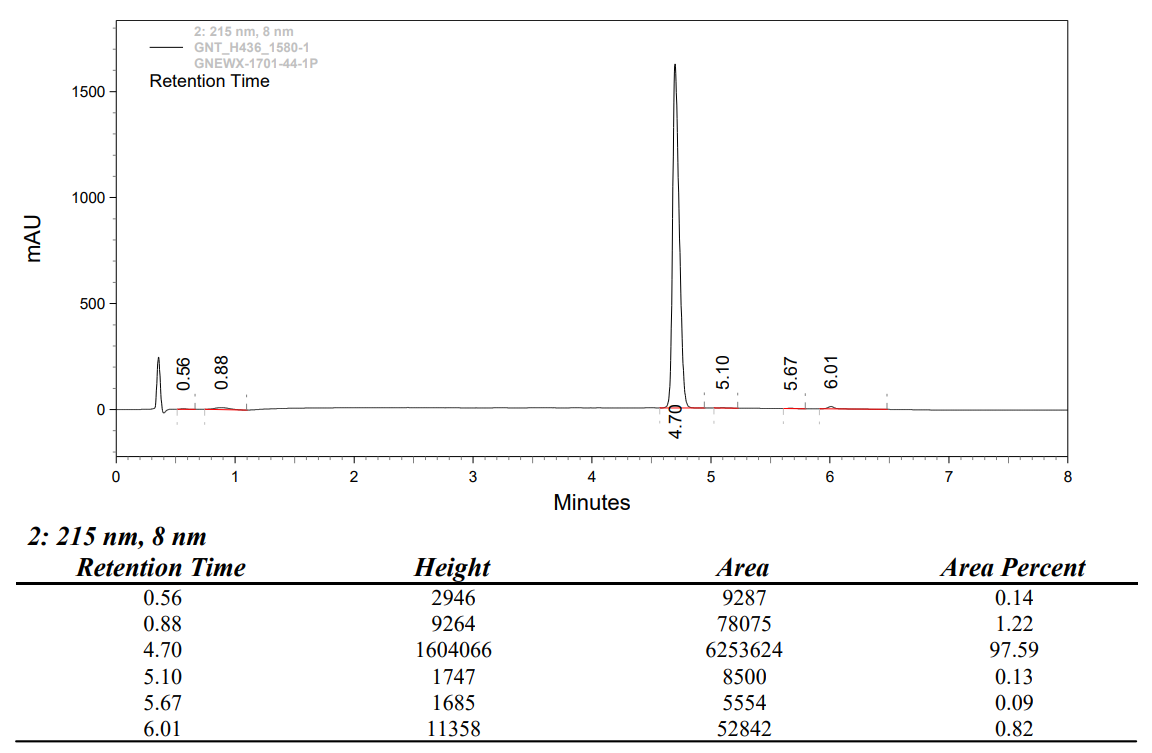


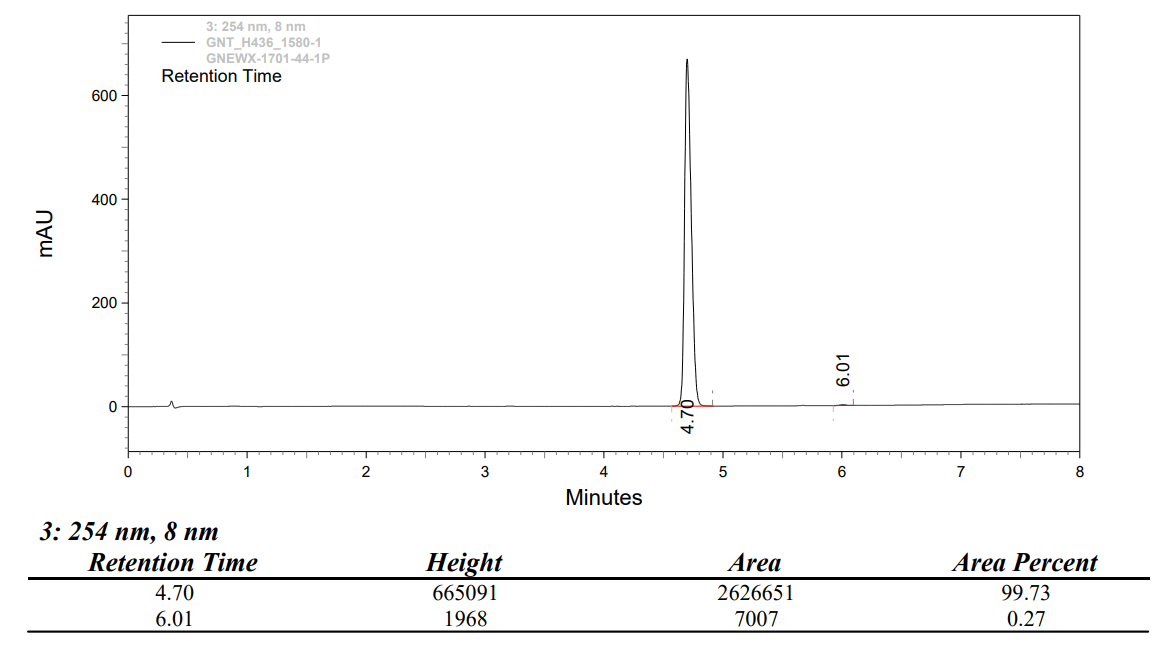


HPLC chromatogram of compound **5**


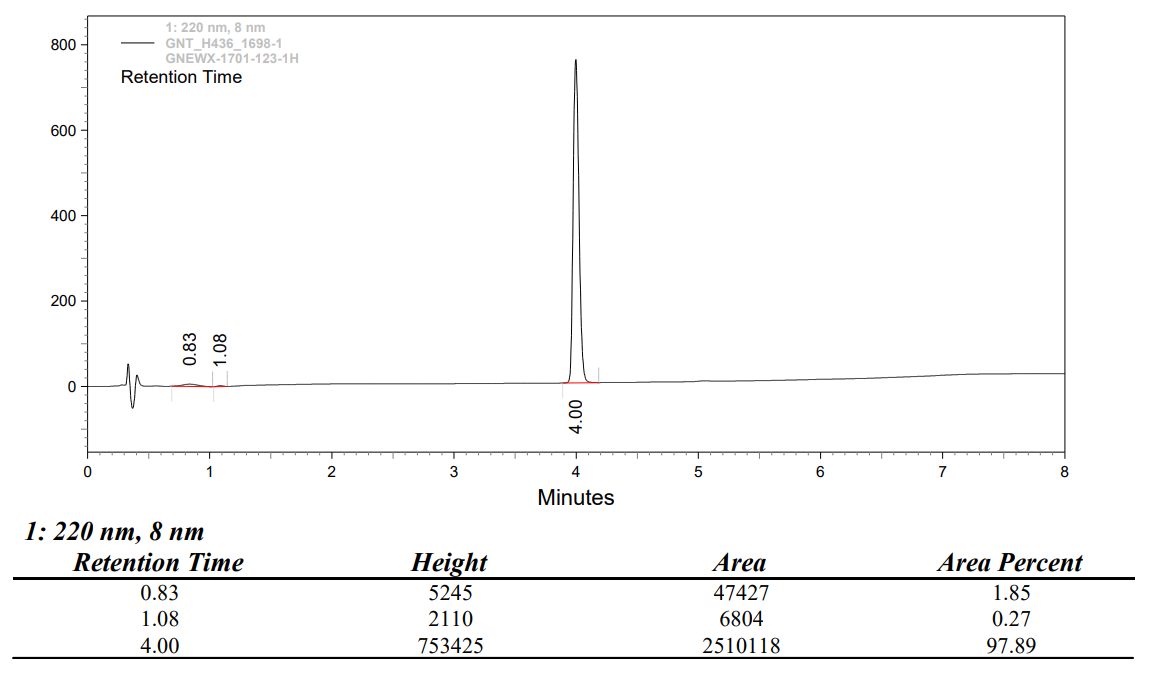


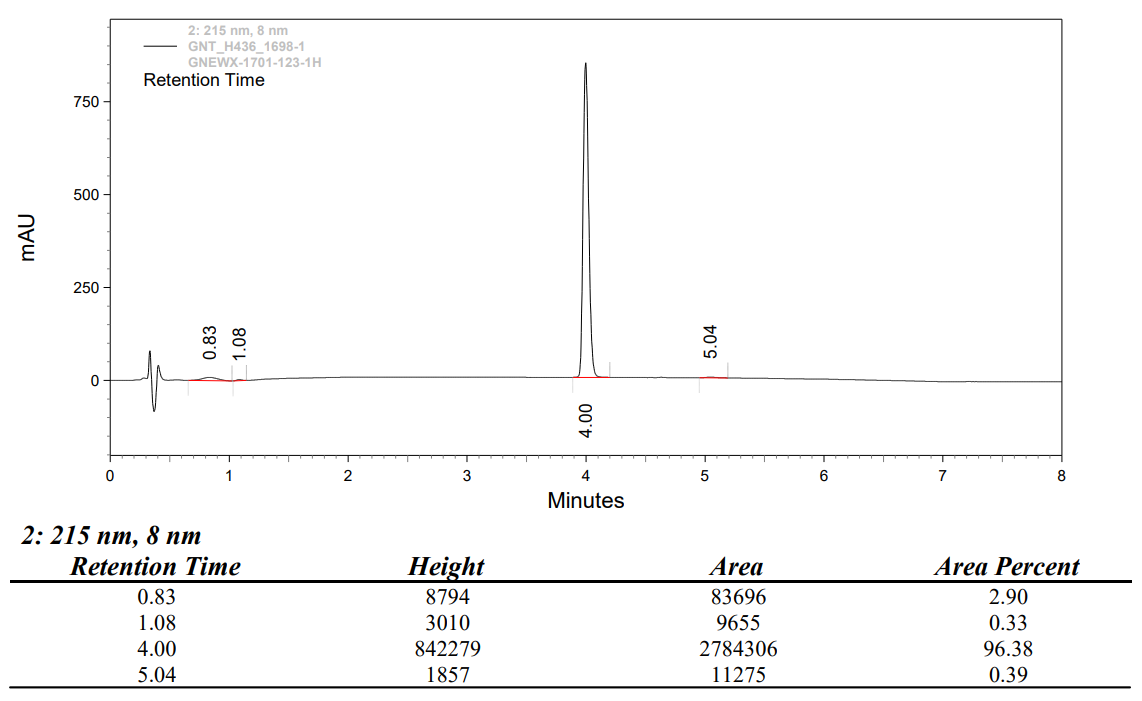


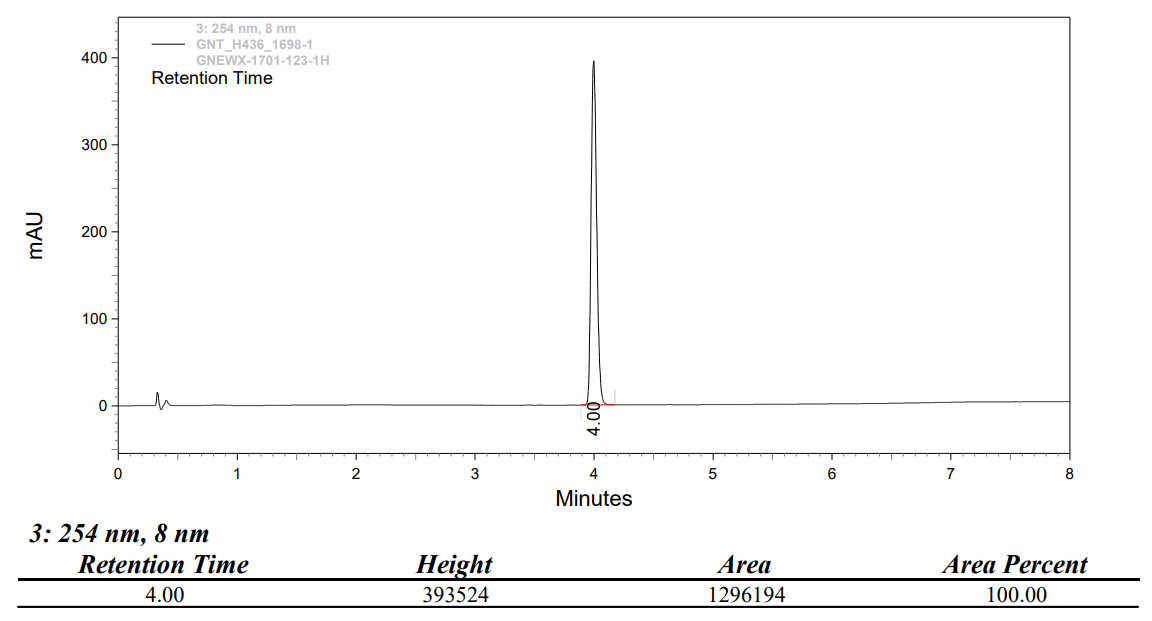

Supplement: Supplementary file 1. — (A) Cryogenic electron microscopy (cryo-EM) data collection, refinement and validation statistics. (B) NaV subtype selectivity for selected hybrid compounds. [file elife-84151-supp1.docx]
